# Performance of a Deep Learning Reconstruction Method on Clinical Chest–Abdomen–Pelvis Scans from a Dual-Layer Detector CT System

**DOI:** 10.3390/tomography11090094

**Published:** 2025-08-25

**Authors:** Christopher Schuppert, Stefanie Rahn, Nikolas D. Schnellbächer, Frank Bergner, Michael Grass, Hans-Ulrich Kauczor, Stephan Skornitzke, Tim F. Weber, Thuy D. Do

**Affiliations:** 1 Department of Diagnostic and Interventional Radiology, Medical Center, Faculty of Medicine, University of Freiburg, 79106 Freiburg, Germany; 2 Clinic of Diagnostic and Interventional Radiology, Heidelberg University Hospital, 69120 Heidelberg, Germany; 3 Translational Lung Research Center Heidelberg (TLRC), German Center for Lung Research (DZL), Heidelberg University Hospital, 69120 Heidelberg, Germany; 4 Philips GmbH Innovative Technologies, 22335 Hamburg, Germany

**Keywords:** computed tomography, deep learning reconstruction, image quality, image noise, denoising

## Abstract

*Objective*: The objective of this study was to compare the performance and robustness of a deep learning reconstruction method against established alternatives for soft tissue CT image reconstruction. *Materials and Methods*: Images were generated from portal venous phase chest–abdomen–pelvis CT scans (*n* = 99) acquired on a dual-layer spectral detector CT using filtered back projection, iterative model reconstruction (IMR), and deep learning reconstruction (DLR) with three parameter settings, namely ‘standard’, ‘sharper’, and ‘smoother’. Experienced raters performed a quantitative assessment by considering attenuation stability and image noise levels in ten representative structures across all reconstruction methods, as well as a qualitative assessment using a four-point Likert scale (1 = poor, 2 = fair, 3 = good, 4 = excellent) for their overall perception of ‘smoother’ DLR and IMR images. One scan was excluded due to cachexia, which limited the quantitative measurements. *Results*: The inter-rater reliability for quantitative measurements ranged from moderate to excellent (*r* = 0.63–0.96). Attenuation values did not differ significantly between reconstruction methods except for DLR against IMR in the psoas muscle (mean + 3.0 HU, *p* < 0.001). Image noise levels differed significantly between reconstruction methods for all structures (all *p* < 0.001) and were lower than FBP with any DLR parameter setting. Image noise levels with ‘smoother’ DLR were predominantly lower than or equal to IMR, while they were higher with ‘standard’ DLR and ‘sharper’ DLR. The ‘smoother’ DLR images received a higher mean rating for overall image quality than the IMR images (3.7 vs. 2.3, *p* < 0.001). *Conclusions*: ‘Smoother’ DLR images were perceived by experienced readers as having improved quality compared to FBP and IMR while also exhibiting objectively lower or equivalent noise levels.

## 1. Introduction

Computed tomography (CT) utilizes ionizing radiation to generate medical images. Radiation exposure to the patient can be controlled through a variety of acquisition parameters, a process that generally follows the ALARA principle (as low as reasonably achievable). Radiologic technicians and radiologists will typically choose a combination of tube voltage (kV)—which correlates approximately quadratically with radiation dose—and a tube current-time product (mAs)—which correlates linearly with radiation dose—suitable for yielding adequate image quality while minimizing patient radiation exposure along with its adverse stochastic and deterministic effects. Depending on the patient’s body composition and the indication of the CT examination, imaging is generally performed in the range of 80–140 kV and 25–250 mAs. While lowering either parameter is an effective way of reducing radiation dose and additional technologies such as tube current modulation enable automated adjustments, dose reduction in any kind will also increase the severity of image noise levels and artifacts in the resulting images, which may impede their diagnostic value [1]. However, image noise can effectively be counteracted by modern reconstruction algorithms, which include iterative, model-based iterative, and hybrid iterative reconstruction algorithms [2,3]. The drawbacks of iterative reconstruction, although dependent on the specific method, comprise long computation times and a ‘patchy’ image appearance. This motivated the development of deep learning reconstruction (DLR) algorithms which take advantage of complex multilayer neural networks [3,4].

Current research indicates that DLR algorithms, with a suitable network architecture and adequate training, may provide an effective means for lowering image noise levels in clinical CT data. Phantom studies have demonstrated that they are capable of elevating the image quality from noise-rich, reduced-dose CT acquisitions closer to the level of standard-dose acquisitions [5,6,7,8]. Other studies used clinical examinations to assess the diagnostic usability of the DLR images and based their assessment on quantitative and qualitative findings, including measured image noise levels in specific organs or diagnostic confidence regarding organ lesions [9,10,11,12,13]. While these studies considered multiple deep learning network architectures and data acquired on different scanner types—either conventional single-source or dual-source CT scanners—for their overall positive findings, a comprehensive study of the performance of a DLR algorithm on data from a dual-layer detector CT scanner has not yet been conducted.

Therefore, the objective of this study was to test the hypothesis that a deep learning reconstruction algorithm with adaptive, user-defined noise reduction capability would improve the image quality of clinical chest–abdomen–pelvis scans from a dual-layer detector CT system over traditional reconstruction methods.

## 2. Materials and Methods

### 2.1. Patient Sample and Image Acquisition

**Figure 1:**
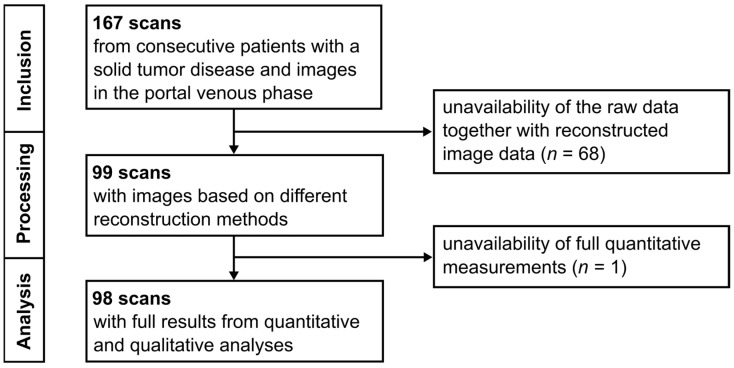
Flow diagram illustrating the inclusion and exclusion of CT scans during the phases of consecutive inclusion, data processing, and image analysis.

The local radiology information system was continuously screened over a seven-month period for newly acquired contrast-enhanced chest–abdomen–pelvis CT examinations performed on a dual-layer detector scanner (IQon Spectral CT, Philips Healthcare, Amsterdam, The Netherlands). Patients referred for staging of solid tumor disease and examined using a scan protocol that included a portal venous phase were consecutively included in the sample. This yielded a database of 167 examinations, with repeat imaging allowed at varying staging intervals for individual patients. Examinations were excluded if raw data and corresponding reconstructed image data were not fully available, or if the complete set of quantitative measurements outlined in Section 2.3: Image Assessment could not be performed. The final study sample comprised 98 examinations with portal venous phase images from 93 patients (Figure 1). The mean patient age at the time of imaging was 64 years. The minimum time between examinations from identical patients was 84 days.

All of the chest–abdomen–pelvis scans were performed with the same image acquisition protocol. The full patient characteristics and scan parameters are compiled in Table 1.

### 2.2. Image Reconstruction

The raw and clinically reconstructed image data were pseudonymized for off-site processing, which comprised reconstructing the raw data sets with different algorithms while retaining basic settings from the clinical images, such as field of view, slice thickness, and slice increment. The algorithms included a reference reconstruction (IMR), a pure FBP reconstruction, and a DLR algorithm, which internally utilizes a trained neural network model based on a deep learning architecture for image reconstruction. Image reconstruction was performed by computing three different types of reconstructions per given raw data acquisition. First, a standard filtered back projection (FBP) reconstruction was used as a baseline image, without any further iterative or other noise reduction algorithms or DLR algorithms applied. Second, every acquisition was processed using an iterative reconstruction algorithm, namely knowledge-based iterative model reconstruction (IMR) at Level 1 (Philips Healthcare, Amsterdam, The Netherlands) for soft tissue protocols. Third, each acquisition was reconstructed using a DLR method with three different denoising presets, which were ‘standard’, ‘sharper’, and ‘smoother’. These presets controlled the denoising strength of the employed deep learning algorithm based on an internal parameter setting. All images were reconstructed using a slice thickness of 1.5 mm and a slice increment of 0.75 mm. Image reconstruction was completed successfully for all 99 examinations.

The DLR algorithm used in this study is a vendor-supplied research prototype (Philips Healthcare, Amsterdam, The Netherlands). It is based on a deep learning neural network architecture, which is trained in a supervised manner: the network is presented with low-dose image samples and its task is to replicate the image appearance of routine-dose FBP image reconstructions [14]. The resulting DLR algorithm is becoming robust with respect to a variety of inherent variations encountered in CT image acquisitions. The matched low-dose and routine-dose training images for the supervised CNN training are created by an advanced low-dose simulation technique which accounts for both photon and electronic noise [15]. The mean squared error is used to optimize the network parameters with the so-called adaptive moment estimation optimizer (ADAM) [16]. In inference mode, layers and weights of the trained network are fixed to enable fast clinical reconstruction workflows. Different DLR settings control the noise reduction strength in the final output images based on user selection. The deep learning part of this reconstruction chain has previously been used in conjunction with a single-layer energy-integrating detector CT scanner and was modified for this research to be used with a dual-layer detector scanner, such as the one employed in this study [8,13]. None of the CT acquisitions used within this study cohort were included in the training or validation datasets of the DLR algorithm under investigation, nor were they employed for fine-tuning or any other form of model adjustment. Consequently, the performance evaluation of the DLR algorithm, as tested within the scope of this study, constitutes an unbiased assessment on unseen test data.

### 2.3. Image Assessment

**Figure 2:**
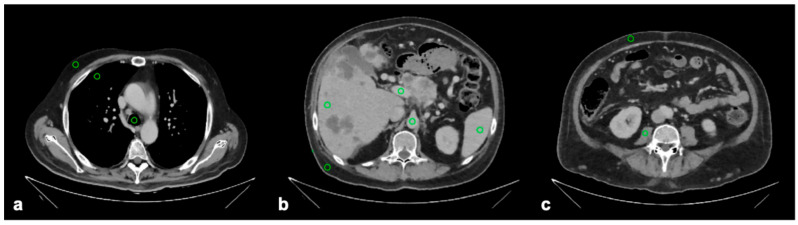
Regions of interest (green circles) as placed on (**a**) thoracic, (**b**) upper abdominal, and (**c**) lower abdominal cross-sections for measurements of image attenuation and image noise. The regions of interest measured 50 mm^2^ and covered the following structures: tracheal lumen, lung tissue, hepatic tissue, splenic tissue, portal vein, abdominal aorta at the height of the superior mesenteric artery, psoas muscle, and at each of the three levels, subcutaneous fat.

A quantitative image assessment was performed independently by two board-certified radiologists with ten and eleven years of professional experience. Using the portal venous phase images, regions of interest (ROIs) were placed in ten anatomical structures, including the tracheal lumen (as a representative air-filled space), lung tissue, right liver lobe at the height of the portal vein, splenic tissue, portal vein, abdominal aorta at the height of the superior mesenteric artery, psoas muscle at the level of the fourth lumbar vertebra, and subcutaneous fat on the right side of the thorax, the upper abdomen, and the lower abdomen (Figure 2). The selection of structures was based on clinically relevant organs or regions, as well as on sites of physical interest examined in previous studies to enable comparability. All ROIs were circular with an area size of 50 mm^2^ and were copied between the different image series (FBP, IMR, and DLR) to maintain comparability and reproducibility. Attenuation was quantified as the mean value within each ROI, whereas image noise was defined as the corresponding standard deviation. The signal-to-noise ratio (SNR) was then computed as the ratio of absolute attenuation to noise.

A qualitative image assessment was performed independently by two board-certified radiologists with six and eleven years of professional experience while blinded to the reconstruction method. The overall image quality of IMR images and ‘smoother’ DLR images (DLR setting chosen based on the results from the quantitative image assessment) from all patients was rated on a 4-point Likert scale (1 = poor; 2 = fair; 3 = good; 4 = excellent) while taking all three body regions (chest, upper abdomen, and lower abdomen) depicted on the portal venous phase images into consideration and placing an emphasis on image noise levels in subcutaneous fat, the viscera, large blood vessels, the presence of image blur, and the conspicuity of organ lesions, especially if tumor-associated. A standard soft tissue windowing level of 40 HU and a width of 400 HU were used.

Results from the quantitative and qualitative analyses were produced for 98 examinations (99%) because in one case, advanced cachexia prevented quantitative measurements in subcutaneous fat, and the case was consequently excluded.

### 2.4. Statistics

Statistical analysis was conducted in R version 4.2.0 (The R Foundation) and SAS version 9.4 (SAS Institute Inc., Cary, NC, USA). All examinations in the final study sample were treated as independent observations. Descriptive statistics were determined by the mean and standard deviation or the median and interquartile range for continuous variables and as counts and percentages for categorical variables. The normal distribution of the data was assessed by an evaluation of histograms, Q-Q-plots, and standardized values of skew and kurtosis. The inter-rater variability of ROI measurements was assessed by calculating the intraclass correlation (ICC) coefficients with corresponding 95% confidence intervals (CIs) for attenuation measurements on FBP images. The estimations of the former were based on single-rater [k = 2], absolute agreement, 2-way random effects models, and a bootstrapping approach was used for the latter. Comparisons of quantitative image quality parameters across reconstruction methods were conducted using a one-way repeated-measures ANOVA, and in case of a significant main effect, the Tukey post hoc test was additionally performed. Mean ratings for image series from each reconstruction method were calculated across readers and compared using the paired samples *t*-test. Additionally, the individual readers’ preferred reconstruction method was determined. The level of statistical significance was set to *p* < 0.05.

## 3. Results

### 3.1. Quantitative Image Quality Assessment

The inter-rater reliability across the ten measurement locations ranged from moderate to excellent (*r* = 0.63–0.96, Table 2). The quantitative results are summarized in Table 3 and Table 4, complemented by Supplemental Figure S1 and Supplemental Table S1. The DLR images showed a high stability of attenuation values against FBP and IMR images, as significant differences within structures were not observed except for the psoas muscle, where the mean attenuation values in DLR images differed up to +3.0 HU (+5.2%) from IMR images (*p* < 0.001) depending on the DLR parameter setting. Notably, no significant difference was observed in the corresponding comparison of DLR against FBP.

However, for image noise levels, significant differences between reconstruction methods were observed for all structures: mean image noise levels in the DLR images were consistently lower than in FBP images regarding all structures and using any parameter setting (*p* = 0.01 to *p* < 0.001 in global testing). These differences remained significant in post hoc testing except for the lung. For the ‘smoother’ DLR images, the mean image noise levels were also predominantly lower than or equal to those in IMR images, which included reductions of −14.1% and −16.9% in the liver and spleen, respectively, and −2.9% to −7.0% in the subcutaneous fat of different body regions, although these differences were not at the level of statistical significance in post hoc testing. This was contrasted by higher mean image noise levels in the trachea, lung, aorta, and psoas muscle (up to +15.7%), although only in the trachea with post hoc significance. For the ‘standard’ DLR and ‘sharper’ DLR images, noise levels were also consistently lower than in FBP images but higher than in IMR images. The SNR was generally highest in ‘smoother’ DLR images, with varying post hoc significance against IMR images (Supplemental Table S1).

### 3.2. Qualitative Image Quality Assessment

**Figure 3:**
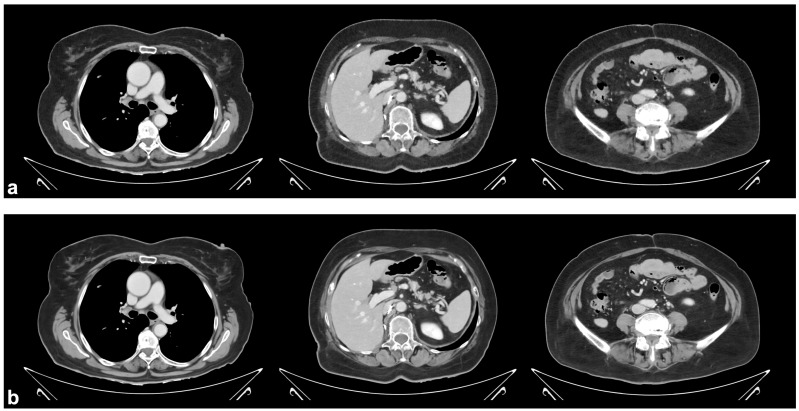
Reconstructed axial images of a CT scan using either the (**a**) IMR reference or the (**b**) ‘smoother’ DLR method. Left to right: chest, upper abdomen, lower abdomen. Window level: 40 HU. Window width: 400 HU. An identical hanging layout was used in the qualitative analysis. IMR: iterative model-based reconstruction. DLR: deep learning reconstruction. HU: Hounsfield unit.

**Figure 4:**
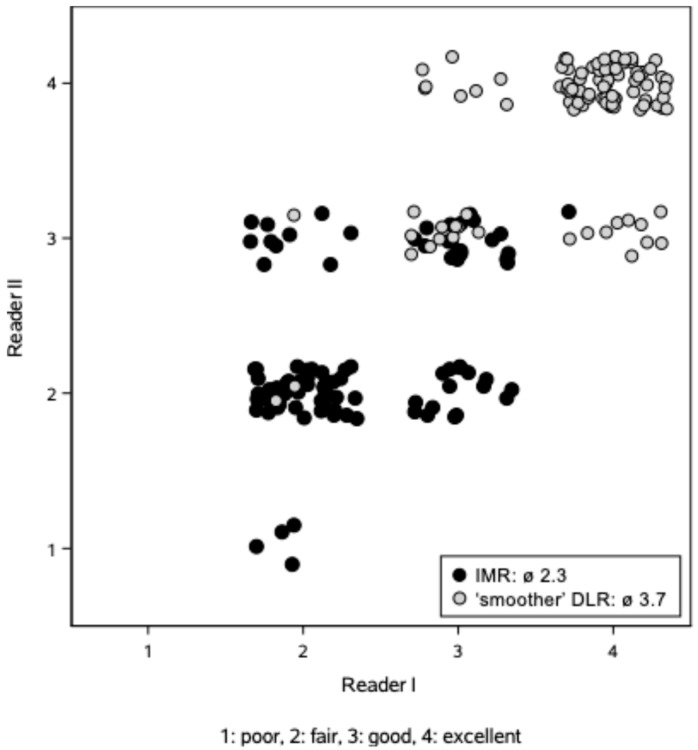
Qualitative ratings from two readers using a four-point Likert scale. The qualitative assessment shows better overall image quality for the ‘smoother’ DLR images compared to the IMR images. ‘Jittering’ avoids overlying data points. IMR: iterative model reconstruction. DLR: deep learning reconstruction.

Examples from the qualitative image quality assessment are shown in Figure 3. The inter-rater reliability was good (*r* = 0.83). The average scores for the IMR images used as reference and the ‘smoother’ DLR images were 2.3 ± 0.5 and 3.7 ± 0.5, respectively (*p* < 0.001, Figure 4), based on the overall image impression by the readers, including image noise, image blur, and organ lesion conspicuity. The two readers preferred the ‘smoother’ DLR images, as seen by higher scores for these in 91 (93%) and 92 (95%) cases, respectively. Among the remaining cases, the Likert scores for the two reconstruction methods given by a reader were either tied, as seen in five (5%) and four (4%) cases, respectively, or the IMR reference reconstruction was preferred.

## 4. Discussion

This retrospective study assessed the performance and robustness of a deep learning reconstruction (DLR) algorithm for computed tomography images (CT) with an adaptive, user-defined noise reduction capability. Results of quantitative and qualitative analyses on 98 clinical portal venous phase chest–abdomen–pelvis CT acquisitions revealed that image noise levels in DLR images were lower than in reference images from filtered back projection (FBP) and, when using the strongest parameter setting, from iterative model reconstruction (IMR); especially regarding the liver, spleen, and subcutaneous fat—although these were not at the level of statistical significance. Additionally, DLR images were rated as superior regarding overall quality perception and preferred over IMR images by two experienced readers.

The measured image noise levels were lowest on IMR images and DLR images reconstructed with the ‘smoother’ setting, that is, the strongest DLR variant used in this study. Differences in image noise levels between these two reconstruction methods were mostly not statistically significant and, if present, limited to a range of−16.9% to +13.6% in terms of ‘smoother’ DLR noise levels against the IMR reference. Nonetheless, the ‘smoother’ DLR images received superior ratings in the qualitative analysis by a significant margin. Thus, ‘smoother’ DLR constituted the best-performing parameter setting and, considering the relevance of image noise levels in the liver, spleen, and fat as opposed to the trachea, lung, aorta, and the psoas muscle for oncological staging through soft tissue images, may be considered the best-performing reconstruction method overall. Similar observations were made by Son et al. [12] in an analysis of image noise levels and sharpness in post-contrast abdominopelvic CT scans from 120 pediatric patients using either a hybrid iterative reconstruction algorithm termed adaptive statistical iterative reconstruction (ASIR-V) with blending factors of 50% and 100% or a DLR method in combination with a single-source CT scanner. They found that the highest strength DLR setting provided the best qualitative ratings, and in their quantitative analysis, limited to measurements in the liver, DLR images showed lower image noise levels than ASIR-V images [12]. Addressing a different imaging domain, Park et al. [11] analyzed image noise levels and sharpness of vessels, liver, and muscle in lower extremity CT angiography of 37 adult patients using the same DLR method as Son et al. and ASIR-V with blending factors of 50% and 100%, also in combination with a single-source CT scanner. The observed quantitative differences in image noise levels between the strongest of three DLR variants and the ASIR-V blends, if statistically significant, were modest and ranged in both directions, varying by anatomical structure. However, these DLR images were rated best in a qualitative analysis regarding their overall quality perception [11]. In the present study, the observed attenuation stability strongly attests to the preserved authenticity of the DLR images. A statistically significant difference between DLR and IMR attenuation values was only observed for the psoas muscle, where the DLR images showed a higher mean attenuation by up to 3.0 HU (5.2%); however, no significant difference was found in testing against the FBP images, showing a better attenuation stability of DLR than IMR in this anatomical structure. A lower stability of DLR attenuation values was observed by Park et al. [11], although the comparison considered only ASIR-V and not FBP. In a phantom study, Greffier et al. also noted reduced noise levels in images reconstructed with a DLR algorithm compared to ASIR-V, depending on the DLR algorithm strength [17]. At the same time, they found the noise texture in the DLR images to appear more natural than in ASIR-V images. Nonetheless, this aspect varied between different DLR algorithms, as shown in another phantom study by the same authors [18]. These findings were mostly seconded by Im et al. for images from a lung phantom reconstructed with another DLR method [19].

None of the aforementioned studies used images that were reconstructed with an iterative reconstruction algorithm from data acquired on a dual-layer detector CT, which was the primary reference method in the present study. Our results were qualitatively in line with their previous findings, which positively supports our initial hypothesis. While we were unable to assess DLR performance on scans acquired with reduced radiation doses in our study, the findings suggest that it carries the potential for further dose reduction by effectively counteracting image quality degradation from increased noise. This possibly includes current low-dose protocols as well and could make, among others, low-dose chest CT a suitable cohort for testing this hypothesis and its implications for clinical imaging and prospective screening programs. Moreover, the development of a DLR method for use with a dual-layer detector CT scanner, characterized by ‘always on’ spectral information collection, provides an opportunity to evaluate a fully spectral DLR method in a future study. This perspective further substantiates the significance of our current study, which first evaluated a DLR method in a clinical setting for this specific type of CT scanner.

The following limitations of our study deserve consideration: the retrospective data collection impacted the ability to control for unforeseen confounding variables, and the exclusions based on the unavailability of raw data together with reconstructed image data introduced a potential source of selection bias. The restriction to scans in the portal venous contrast phase (while representing the predominant, though not exclusive, phase used for soft tissue assessment), soft tissue kernels, and specific anatomical structures for quantitative analyses limits the generalizability of the results. Despite this, efforts were made to consider regions that are significant for clinical reporting and to maintain comparability and reproducibility of the measurements through a standardized ROI placement by two independent readers. Additional studies will be needed to evaluate the DLR method for other domains, such as lung and bone imaging, and to assess its generalization strength to data from different clinical sites. Furthermore, we acknowledge that standardization in the evaluation of image reconstruction tasks remains challenging and limits cross-study comparability due to different means of analysis. We also did not explicitly evaluate the effects of algorithmic processing on clinical tasks and decision-making such as feature detection, precise lesion measurements, or the derivation of definitive diagnoses. However, the qualitative image assessment explicitly asked readers to, besides image noise, also consider blur effects—a previously described side effect of denoising algorithms [20]—and lesion conspicuity in their overall quality rating, and they preferred the DLR images nonetheless. Adding to this argument is that there is no formal ground truth for these variables, despite iterative reconstruction algorithms being an established reference.

## 5. Conclusions

The evaluated DLR method produced soft tissue images from chest–abdomen–pelvis scans that were perceived by experienced readers as having improved quality relative to conventional FBP and IMR while also exhibiting objectively lower or comparable image noise levels.

## Figures and Tables

**Table 1:** Patient characteristics for all 98 examinations in the final study sample and CT scan parameters for the corresponding portal venous phase images.

Parameter	Value
Sex (female; count and percentage)	47 (48%)
Age (years)	64 ± 12
Height (cm)	172 ± 11
Weight (kg)	77 ± 16
BMI (kg/m^2^)	25 (23–28)
Pitch	0.798
Beam Collimation (mm)	64 × 0.625
Peak Tube Voltage (kV)	120
Tube Current-Time Product (mAs)	74
Dose Modulation	Enabled (attenuation based; dose right index 13)
Predicted CTDI_vol_ (mGy)	7.5 (using a 32 cm body phantom)
Scanner-Reported CTDI_vol_ (mGy)	6.4 (6.0–7.1)
Contrast Medium	Nonionic 350 mgI/mL (Accupaque^TM^ 350, GE Healthcare, Chicago, IL, USA)
Delay after Contrast Medium Injection (s)	60

The data in this table are presented as counts with percentages, mean ± standard deviation, median (interquartile range), or individual values. All images were acquired using a dual-layer detector CT scanner (IQon Spectral CT, Philips Healthcare, Amsterdam, The Netherlands). CTDI_vol_: volumetric computed tomography dose index.

**Table 2:** Inter-rater variability for attenuation measurements from portal venous phase images in the quantitative analysis as assessed by ICC.

Region of Interest	ICC [95% CI]
Trachea (Air)	0.63 [0.50, 0.74]
Lung	0.80 [0.72, 0.86]
Liver	0.95 [0.92, 0.96]
Spleen	0.94 [0.92, 0.96]
Portal Vein	0.93 [0.90, 0.95]
Aorta	0.94 [0.91, 0.96]
Psoas Muscle	0.63 [0.49, 0.73]
Thoracic Fat	0.88 [0.83, 0.92]
Upper Abdominal Fat	0.96 [0.94, 0.97]
Lower Abdominal Fat	0.90 [0.85, 0.93]

The measurements were performed independently by two observers. Inter-rater variability was assessed on FBP images. ICC: intra-class correlation coefficient. CI: confidence interval. FBP: filtered back projection.

**Table 3:** Quantitative analysis of image attenuation.

Region of Interest	Attenuation (HU)					*p*
	FBP	IMR	‘Smoother’ DLR	‘Standard’ DLR	‘Sharper’ DLR	
Trachea (Air)	−993.8 ± 6.5	−994.7 ± 6.3	−994.4 ± 6.9	−994.6 ± 6.9	−994.5 ± 6.8	0.67
Lung	−873.2 ± 35.6	−874.0 ± 34.5	−872.9 ± 36.1	−873.8 ± 35.6	−874.0 ± 35.5	0.99
Liver	103.3 ± 16.3	102.1 ± 16.2	103.0 ± 16.1	102.9 ± 16.2	102.7 ± 16.1	0.97
Spleen	105.5 ± 18.5	103.9 ± 18.0	105.1 ± 18.0	105.2 ± 18.3	104.9 ± 18.3	0.93
Portal Vein	146.3 ± 25.0	144.9 ± 25.0	146.8 ± 24.7	146.6 ± 24.8	145.8 ± 24.7	0.95
Aorta	141.7 ± 23.0	139.7 ± 23.0	141.7 ± 22.6	141.7 ± 22.8	141.1 ± 22.7	0.89
Psoas Muscle	61.5 **^b^** ± 7.7	58.0 **^a^** ± 7.2	61.0 **^b^** ± 7.1	60.9 **^b^** ± 7.2	61.0 **^b^** ± 7.3	**<0.001**
Thoracic Fat	−105.5 ± 14.6	−106.5 ± 14.5	−105.8 ± 14.5	−105.9 ± 14.5	−106.0 ± 14.5	0.98
Upper Abdominal Fat	−98.7 ± 19.5	−99.9 ± 19.4	−99.1 ± 19.3	−97.8 ± 24.8	−99.3 ± 19.5	0.90
Lower Abdominal Fat	−105.9 ± 13.9	−106.8 ± 13.1	−105.9 ± 13.5	−106.0 ± 13.6	−106.1 ± 13.7	0.96

Data for *n* = 98 examinations are presented as mean ± standard deviation. *p* values were extracted from a one-way repeated-measures ANOVA. For significant *p* values, the superscript letters indicate the results of the Tukey post hoc test (means with the same letter are not significantly different) and their alphabetical order denotes increasing mean values. HU: Hounsfield unit. FBP: filtered back projection. IMR: iterative model reconstruction. DLR: deep learning reconstruction. Bold: Used to mark values as statistically significant.

**Table 4:** Quantitative analysis of image noise.

Region of Interest	Noise (HU)								*p*
	FBP	IMR	‘Smoother’ DLR		‘Standard’ DLR		‘Sharper’ DLR		
Trachea (Air)	16.2 **^e^** ± 3.5	5.1 **^a^** ± 1.6	5.9 **^b^** ± 2.1	(+15.7%)	8.7 **^c^** ± 2.1	(+70.6%)	12.4 **^d^** ± 2.7	(+143.1%)	**<0.001**
Lung	37.4 **^b^** ± 18.4	31.0 **^a^** ± 18.8	32.5 **^ab^** ± 20.0	(+4.8%)	33.6 **^ab^** ± 19.2	(+8.4%)	35.3 **^ab^** ± 18.7	(+13.9%)	**0.01**
Liver	31.4 **^d^** ± 6.3	6.4 **^a^** ± 1.0	5.5 **^a^** ± 2.3	(−14.1%)	13.6 **^b^** ± 2.8	(+112.5%)	22.0 **^c^** ± 4.4	(+243.8%)	**<0.001**
Spleen	33.3 **^d^** ± 9.4	7.1 **^a^** ± 4.7	5.9 **^a^** ± 3.0	(−16.9%)	14.3 **^b^** ± 3.8	(+101.4%)	23.0 **^c^** ± 5.8	(+223.9%)	**<0.001**
Portal Vein	41.7 **^d^** ± 8.5	8.3 **^a^** ± 1.4	8.3 **^a^** ± 2.8	(±0.0%)	18.5 **^b^** ± 3.7	(+122.9%)	29.3 **^c^** ± 5.9	(+253.0%)	**<0.001**
Aorta	45.2 **^d^** ± 8.6	8.2 **^a^** ± 1.5	8.4 **^a^** ± 3.4	(+2.4%)	19.6 **^b^** ± 3.8	(+139.0%)	31.3 **^c^** ± 5.9	(+281.7%)	**<0.001**
Psoas Muscle	44.4 **^d^** ± 10.1	7.2 **^a^** ± 1.7	7.6 **^a^** ± 3.1	(+5.6%)	18.1 **^b^** ± 4.5	(+151.4%)	28.9 **^c^** ± 7.0	(+301.4%)	**<0.001**
Thoracic Fat	16.8 **^d^** ± 3.8	5.2 **^a^** ± 1.5	4.9 **^a^** ± 2.4	(−5.8%)	8.4 **^b^** ± 2.1	(+61.5%)	12.5 **^c^** ± 2.7	(+140.4%)	**<0.001**
Upper Abdominal Fat	24.4 **^d^** ± 6.1	6.8 **^a^** ± 2.1	6.6 **^a^** ± 3.2	(−2.9%)	11.9 **^b^** ± 3.3	(+75.0%)	17.8 **^c^** ± 4.3	(+161.8%)	**<0.001**
Lower Abdominal Fat	25.3 **^d^** ± 7.2	5.7 **^a^** ± 1.5	5.3 **^a^** ± 2.5	(−7.0%)	11.2 **^b^** ± 2.9	(+96.5%)	17.4 **^c^** ± 4.2	(+205.3%)	**<0.001**

Data for *n* = 98 examinations are presented as mean ± standard deviation and complemented by percentage changes from IMR to DLR. *p* values were extracted from a one-way repeated-measures ANOVA. For significant *p* values, the superscript letters indicate the results of the Tukey post hoc test (means with the same letter are not significantly different), and their alphabetical order denotes increasing mean values. HU: Hounsfield unit. FBP: filtered back projection. IMR: iterative model reconstruction. DLR: deep learning reconstruction. Bold: Used to mark values as statistically significant.

## Data Availability

The measurement data generated during this study are not publicly available but are available from the corresponding author upon reasonable request. The imaging data are not available due to ethical and legal considerations.

## Supplementary Materials

The following supporting information can be downloaded at: https://www.mdpi.com/article/10.3390/tomography11090094/s1. Figure S1: Image noise in ten anatomical structures across five different soft-tissue reconstruction algorithms. Table S1: Quantitative analysis of image signal-to-noise ratios.

## Abbreviations

ADAM	adaptive moment estimation optimizer
ALARA	as low as reasonably achievable
CI	confidence interval
CT	computed tomography
CTDI_vol_	volumetric computed tomography dose index
DLCT	dual-layer spectral detector CT
DLR	deep learning reconstruction
FBP	filtered back projection
HU	Hounsfield unit
ICC	intraclass correlation coefficient
IMR	iterative model reconstruction
ROI	region of interest
SD	standard deviation
SNR	signal-to-noise ratio
