# Supplementary material for: Performance of a Deep Learning Reconstruction Method on Clinical Chest–Abdomen–Pelvis Scans from a Dual-Layer Detector CT System
Source: Tomography. 2025 Aug 25;11(9):94. doi: 10.3390/tomography11090094 (PMC12473457; doi:10.3390/tomography11090094)
Supplement: Supplementary file 1 [file tomography-11-00094-s001.zip › tomography-3754597-supplementary.pdf]

Supplementary Materials

Supplemental Figure S1. Image noise in ten anatomical structures across five different soft-tissue reconstruction algorithms.

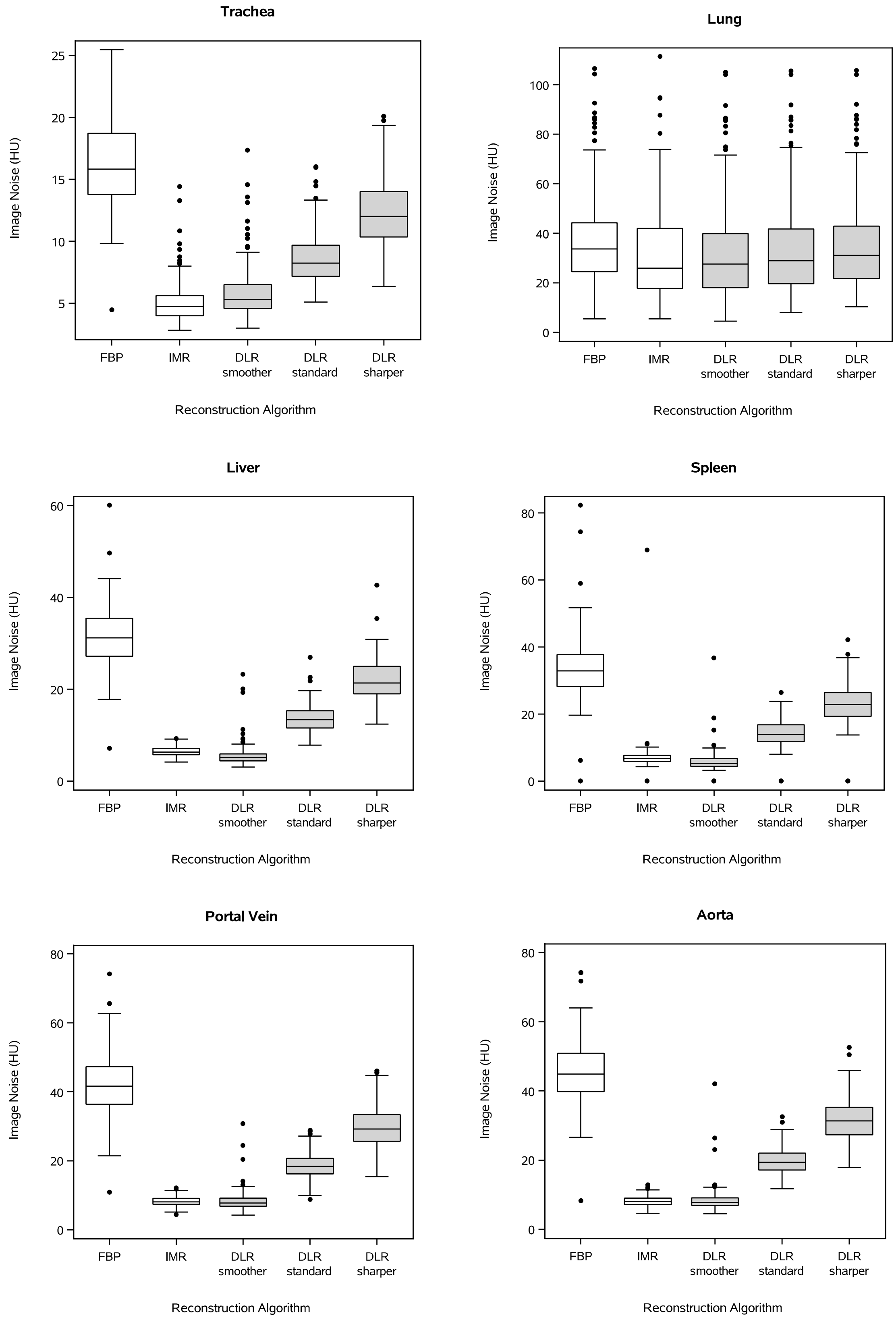

Supplemental Figure S1. (continued)

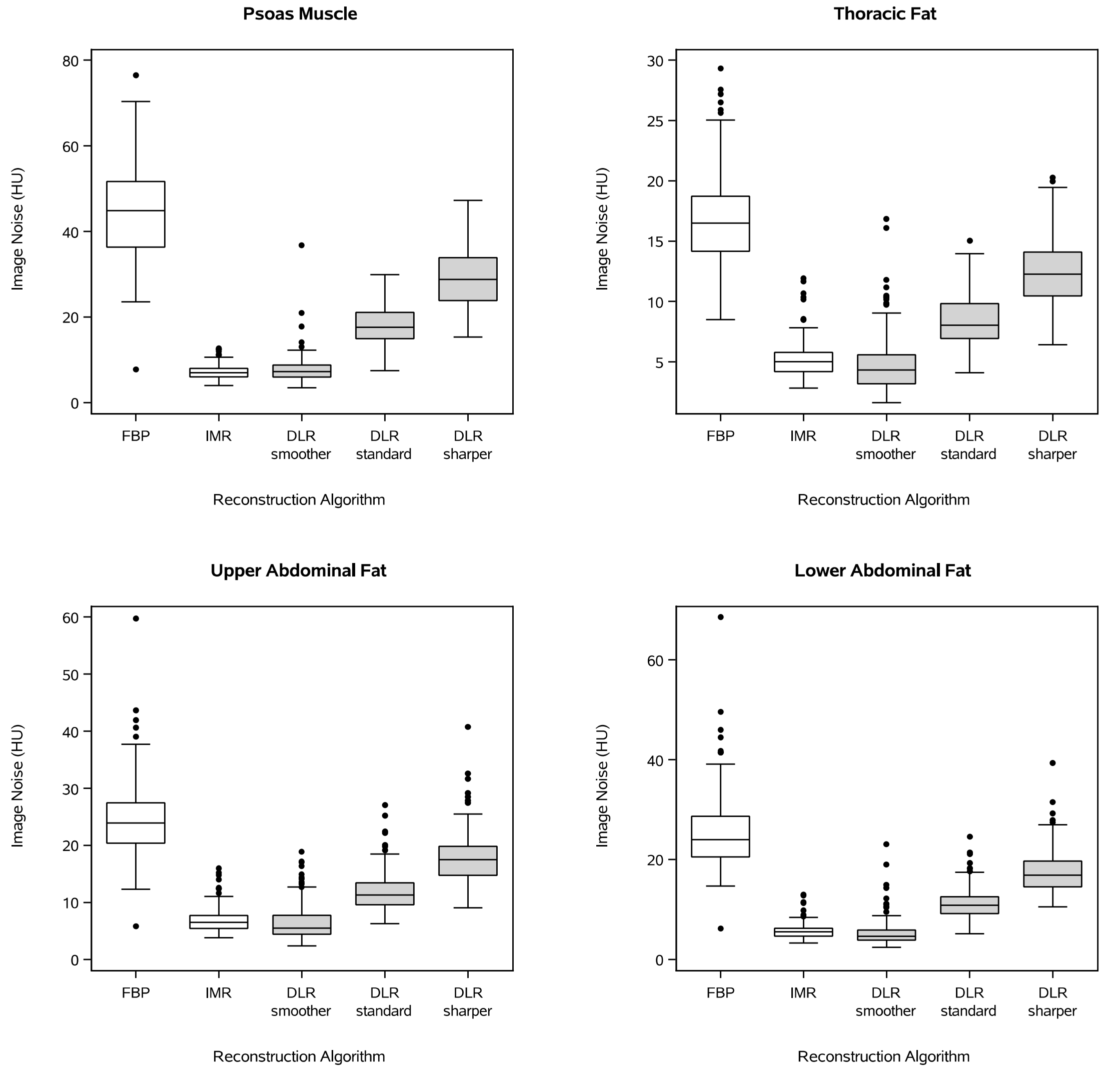

Data for *n* = 98 examinations are presented as Box-Whisker-Plots. *HU: Hounsfield unit. FBP: filtered back projection. IMR: iterative model reconstruction. DLR: deep learning reconstruction.*

Supplemental Table S1. Quantitative analysis of image signal-to-noise ratios.

Region of Interest	SNR					p
	FBP	IMR	’smoother’ DLR	‘standard’ DLR	’sharper’ DLR	
Trachea (Air)	$64.5^{a} \pm 17.7$	$210.3^{e} \pm 52.8$	$185.6^{d} \pm 49.9$	$120.6^{c} \pm 26.2$	$84.2^{b} \pm 18.2$	**< 0.001**
Lung	$29.0^{a} \pm 15.7$	$40.7^{c} \pm 27.1$	$37.9^{bc} \pm 24.4$	$34.5^{ab} \pm 18.3$	$31.3^{a} \pm 15.0$	**< 0.001**
Liver	$3.5^{a} \pm 1.1$	$16.3^{d} \pm 3.9$	$20.3^{e} \pm 6.1$	$7.9^{c} \pm 2.2$	$4.9^{b} \pm 1.4$	**< 0.001**
Spleen	$3.4^{a} \pm 1.2$	$15.8^{d} \pm 4.1$	$19.8^{e} \pm 6.3$	$7.8^{c} \pm 2.3$	$4.8^{b} \pm 1.4$	**< 0.001**
Portal Vein	$3.7^{a} \pm 1.2$	$18.1^{d} \pm 4.9$	$19.1^{d} \pm 6.3$	$8.3^{c} \pm 2.6$	$5.2^{b} \pm 1.7$	**< 0.001**
Aorta	$3.3^{a} \pm 1.1$	$17.7^{d} \pm 4.7$	$18.1^{d} \pm 5.0$	$7.5^{c} \pm 2.1$	$4.7^{b} \pm 1.3$	**< 0.001**
Psoas Muscle	$1.5^{a} \pm 0.6$	$8.5^{d} \pm 2.4$	$8.8^{d} \pm 2.9$	$3.6^{c} \pm 1.1$	$2.2^{b} \pm 0.6$	**< 0.001**
Thoracic Fat	$6.6^{a} \pm 1.8$	$22.1^{d} \pm 6.5$	$26.6^{e} \pm 12.2$	$13.5^{c} \pm 4.1$	$8.9^{b} \pm 2.4$	**< 0.001**
Upper Abdominal Fat	$4.4^{a} \pm 1.7$	$16.3^{d} \pm 5.5$	$18.8^{e} \pm 9.1$	$9.1^{c} \pm 2.9$	$6.0^{b} \pm 1.8$	**< 0.001**
Lower Abdominal Fat	$4.5^{a} \pm 1.6$	$19.8^{d} \pm 5.1$	$23.3^{e} \pm 8.5$	$10.1^{c} \pm 2.7$	$6.4^{b} \pm 1.6$	**< 0.001**

Data for $n = 98$ examinations are presented as mean ± standard deviation. *P* values were extracted from a one-way repeated-measures ANOVA. For significant *p* values, the superscript letters indicate the results of the Tukey post hoc test (means with the same letter are not significantly different) and their alphabetical order denotes increasing mean values. *HU: Hounsfield unit. FBP: filtered back projection. IMR: iterative model reconstruction. DLR: deep learning reconstruction. SNR: signal-to-noise ratio.*
